# The impact of anxiety on canine heart function: a study using echocardiographic techniques

**DOI:** 10.1186/s12917-025-05074-3

**Published:** 2025-10-14

**Authors:** Delara Soghyani, Farnoosh Arfaee, Mohammad Molazem, Reihaneh Soflaei, Arman Abdous

**Affiliations:** 1 Department of Clinical Science, SR.C, Islamic Azad University, Tehran, Iran; 2 Department of Veterinary Surgery and Diagnostic Imaging, Faculty of Veterinary Medicine, University of Tehran, Tehran, Iran; 3 Faculty of Veterinary Medicine, Karaj Branch, Islamic Azad University, Karaj, Iran

**Keywords:** Anxiety, Canine heart function, Echocardiography, Behavioral assessment

## Abstract

Canine anxiety is increasingly recognized in veterinary practice, yet its cardiovascular effects remain poorly defined. This study evaluated 36 anxious dogs and 20 healthy controls using the validated Canine Behavioral Assessment and Research Questionnaire (C-BARQ) and standardized echocardiography. Significant associations were identified between anxiety subtypes and cardiac parameters: general anxiety correlated with increased fractional shortening (FS%; *r* = 0.663, *p* = 0.001), phonophobia with larger left ventricular internal dimension in diastole (LVIDd; *r* = 0.681, *p* < 0.001), and aggression with a higher left atrial-to-aortic root ratio (LA/Ao; *r* = 0.475, *p* = 0.008). Rhythm irregularities were suspected in nearly half of anxious dogs, though classification was limited without ECG confirmation. By contrast, correlations in controls were weak and non-significant.These results provide novel evidence that chronic behavioral anxiety is associated with measurable echocardiographic alterations in dogs, paralleling mechanisms described in human psychocardiology. Incorporating behavioral screening into routine veterinary practice may support early identification and monitoring of dogs at risk for anxiety-related cardiac changes. Larger longitudinal studies with matched controls, ECG verification, and size-indexed parameters are warranted to confirm causality and guide preventive care strategies.

**Supplementary Information:**

The online version contains supplementary material available at 10.1186/s12917-025-05074-3.

## Introduction

Anxiety in dogs is an increasingly recognized concern in veterinary medicine, affecting a significant portion of the canine population [1]. Conditions such as separation anxiety, noise phobia, and generalized fear can lead to behavioral disturbances and reduce quality of life. Emotional stress in animals has been associated with various physiological consequences, including gastrointestinal disorders and compromised immune function; however, its specific impact on cardiovascular health remains underinvestigated [2, 3]. 

In contrast, a large body of human research links anxiety to cardiovascular dysfunction. Chronic anxiety in people is associated with altered heart rate variability, heightened sympathetic tone, and structural changes such as diastolic dysfunction and atrial enlargement—often detectable through echocardiography. Although parallels between human and canine physiology suggest similar psychocardiological mechanisms may occur in dogs, most veterinary studies have focused on physical stressors like exercise or anesthesia rather than emotional triggers [4, 5]. Consequently, the influence of chronic behavioral anxiety on canine cardiac function, particularly as assessed by advanced non-invasive echocardiographic techniques, remains largely unexplored [3]. 

Canine heart disease itself can arise from congenital abnormalities, acquired degenerative conditions such as mitral valve disease, or myocardial disorders including cardiomyopathies [6, 7]. Clinical signs may include fatigue, coughing, exercise intolerance, and dyspnea. Echocardiography—using B-mode, M-mode, and Doppler modalities—has become a cornerstone of cardiac diagnostics in veterinary practice. It provides quantitative insight into chamber size, systolic function, wall motion, and blood flow dynamics [8]. Commonly evaluated parameters include left ventricular internal dimensions at systole and diastole (LVIDs and LVIDd), ejection fraction (EF%), fractional shortening (FS%), and the left atrial-to-aortic root ratio (LA/Ao) [9]. 

This study was designed to address the knowledge gap by comparing echocardiographic parameters in anxious dogs and clinically healthy, non-anxious controls. By integrating validated behavioral assessment tools with echocardiographic evaluation, we aimed to determine whether chronic emotional stress contributes to subclinical or early-stage cardiovascular changes. Identifying such links could support the incorporation of behavioral screening into cardiac risk assessment, potentially enabling earlier intervention and improved clinical outcomes.

## Methods

### Study design

This observational, cross-sectional study was conducted over a six-month period at the Islamic Azad University, Science and Research Branch. Its objective was to assess whether anxiety-related behaviors are associated with alterations in canine cardiac parameters measured through advanced echocardiographic techniques.Dogs were assigned to either an anxiety group or a control group based on behavioral assessment. The anxiety group included dogs with owner-reported signs such as excessive fear of loud noises, separation-related distress, or generalized nervousness. The control group consisted of clinically healthy, non-anxious dogs. Both groups underwent identical echocardiographic protocols, enabling direct comparison of cardiac function. An overview of the experimental workflow is provided in Supplementary Figure S1.

### Ethical considerations

The study was conducted in accordance with institutional ethical standards and approved by the Ethics Committee of the Islamic Azad University, Science and Research Branch, Tehran. Prior to participation, owners of all dogs in both groups received detailed written and verbal information about the study’s objectives, procedures, and potential risks. Written informed consent was obtained, and participants were assured of their right to withdraw at any time without consequence.All echocardiographic procedures were performed by licensed veterinary professionals with specialized training in cardiology. Humane treatment guidelines for animal research were strictly followed, with gentle handling and procedures designed to minimize stress and ensure animal welfare.

### Study population

A total of 74 dogs were screened at the hospital of the Islamic Azad University, Science and Research Branch (Tehran), and affiliated veterinary clinics. Eighteen were excluded—11 due to current or recent use of psychotropic medications and 7 due to comorbid medical conditions—leaving 56 dogs enrolled. Of these, 36 met the criteria for the anxiety group, while 20 clinically healthy, non-anxious dogs served as controls.The anxious group included dogs aged 1–12 years, representing a diverse mix of breeds and sizes to enhance generalizability. Inclusion criteria required owner-reported signs of anxiety—such as noise sensitivity, separation distress, or nervousness in unfamiliar situations—persisting for at least three months, confirmed through history or clinical documentation. Dogs with prior diagnoses of heart disease were excluded to isolate the effect of behavioral anxiety on cardiac function. Additional exclusion criteria included cardiovascular, neurological, or systemic conditions that might affect echocardiographic results, as well as treatment with medications for anxiety or behavioral disorders (e.g., trazodone, fluoxetine, gabapentin).The control group consisted of 20 dogs free of cardiac or behavioral disorders, with consistently low C-BARQ scores (mean global anxiety score: 0.64 ± 0.18). Their mean age was 4.9 ± 3.0 years (range 1.1–9.7), and body weights ranged from 4.4 to 15.0 kg. To minimize environmental and handling differences, control dogs were drawn from the same clinical population and underwent identical echocardiographic evaluation.

### Behavioral assessment of anxiety

Behavioral anxiety was quantified using an adapted version of the Canine Behavioral Assessment and Research Questionnaire (C-BARQ), a standardized instrument widely applied in clinical behavioral research to characterize emotional reactivity in dogs. Six domains were evaluated: separation anxiety, noise sensitivity (phonophobia), fear, aggression, obsessive-compulsive behaviors, and generalized anxiety. Owners rated the frequency of each behavior on a 5-point Likert scale, where 0 indicated “never,” 1 “rarely,” 2 “sometimes,” 3 “often,” and 4 “always.” Observable signs such as excessive barking, destructiveness when left alone, trembling, hiding, or hypervigilance were used to guide accurate scoring.Following completion of the questionnaire, total anxiety scores were calculated as the unweighted arithmetic mean of responses across the six domains, providing a standardized measure of each dog’s overall anxiety level. Dogs were then stratified into three categories: scores below 1.5 were classified as low anxiety, scores between 1.5 and 2.5 as moderate anxiety, and scores above 2.5 as high anxiety. Control dogs consistently demonstrated low scores across all domains, confirming their non-anxious status.

### Echocardiographic assessment

Echocardiographic examinations were performed by a board-certified veterinary cardiologist using a Philips EPIQ 7 ultrasound system equipped with high-resolution B-mode, M-mode, and Doppler functionalities. A sector-array phased transducer probe with a frequency range of 3.5–8 MHz was used for thoracic imaging, as it provides deep penetration with high temporal resolution. For very small dogs (< 3 kg), higher-frequency probes (>8 MHz) were employed to enhance spatial resolution and minimize measurement error, ensuring consistent image quality across the range of body sizes represented in the study.This imaging platform enabled detailed evaluation of cardiac structure and function, including real-time assessment of ventricular wall motion, valve morphology, and blood flow dynamics. Standard veterinary echocardiographic protocols were followed [10] Key measurements included left ventricular internal dimensions at end-systole (LVIDs) and end-diastole (LVIDd), interventricular septal thickness (IVS), fractional shortening (FS%), and ejection fraction (EF%). These indices are central to evaluating myocardial size, contractile performance, and systolic function. M-mode echocardiography was used for precise dimensional analysis of the left ventricle, B-mode imaging for overall morphology and detection of abnormalities such as wall motion irregularities or chamber dilation, and Doppler for intracardiac blood flow and identification of valvular regurgitation across the mitral, aortic, and tricuspid valves. Together, these methods provided comprehensive insight into both structural and hemodynamic aspects of cardiac performance.All echocardiographic data were recorded systematically and analyzed in relation to behavioral anxiety classifications. Both anxious and control dogs underwent identical assessment protocols during a single clinical visit, allowing for direct comparison of structural and functional parameters and evaluation of the potential influence of emotional stress on cardiac function.

### Statistical analysis

Analyses were conducted using IBM SPSS Statistics v26. Descriptive statistics (means, standard deviations, and frequencies) were used to summarize demographic, behavioral, and echocardiographic data. Linear associations between anxiety scores, age, and cardiac parameters were assessed with Pearson’s correlation coefficients. To control for multiple comparisons, p-values were adjusted using the false discovery rate (FDR), and only associations that remained significant after correction were interpreted. Statistical significance was defined as two-tailed *p* < 0.05. Results are reported with correlation coefficients, 95% confidence intervals, effect sizes, and p-values, with selected associations illustrated graphically. A post-hoc power analysis (α = 0.05) showed that the sample size (36 anxious and 20 control dogs) provided > 80% power to detect between-group differences of medium effect size (Cohen’s d ≥ 0.65) and correlations of *r* ≥ 0.44 within the anxious cases.

## Results

### Demographic and sample characteristics

The anxious group consisted of 36 dogs ranging in age from 1 to 12 years, with the largest proportions being 1 year (19.4%) and 2 years (16.7%). Senior dogs, defined as those aged 7 years or older for small to medium breeds, represented 25% of the anxious cases. Breed diversity was notable: Shiba Inu (22.2%), Maltese (19.4%), Miniature Poodle (16.7%), and Jack Russell Terrier (13.9%) were the most common, while French Bulldog, Terrier, Shih Tzu, and Bichon Frise were also represented, creating a heterogeneous sample of small- to medium-sized companion breeds.

Body-weight distribution in the anxious group was skewed toward very small dogs, with 58.3% weighing less than 3 kg. Dogs in the 3–5 kg and 5–10 kg categories accounted for 19.4% and 13.9% of the group, respectively, while 8.3% weighed between 10 and 15 kg. This pattern likely reflects regional breed preferences and the higher prevalence of anxiety in toy and miniature breeds.

The control group (*n* = 20) consisted of clinically healthy dogs with a mean age of 4.9 ± 3.0 years (range 1.1–9.7). Their body weights ranged from 4.4 to 15.0 kg, and breed distribution is shown in Table 1. In contrast to the anxious group, which was predominantly composed of dogs under 3 kg, the control group displayed a more balanced distribution across weight categories.Age was further examined in relation to both behavioral anxiety scores and echocardiographic variables, with correlation patterns presented in Sect. 3 and summarized in Table 2.

**Table 1:** Demographic characteristics of anxious dogs (*n* = 36) and control dogs (*n* = 20)

Characteristic	Anxious Dogs (*n* = 36)	Control Dogs (*n* = 20)
Age (years)	1 year – 7 (19.4%)	1 year – 3 (15.0%)
	2 years – 6 (16.7%)	2 years – 4 (20.0%)
	3 years – 5 (13.9%)	3 years – 2 (10.0%)
	4 years – 4 (11.1%)	4 years – 5 (25.0%)
	5 years – 3 (8.3%)	5 years – 1 (5.0%)
	6 years – 2 (5.6%)	6 years – 2 (10.0%)
	7–12 years – 9 (25.0%)	7–12 years – 3 (15.0%)
Breed Distribution	Shiba Inu – 8 (22.2%)	Maltese – 3 (15.0%)
	Maltese – 7 (19.4%)	Shih Tzu – 2 (10.0%)
	Miniature Poodle – 6 (16.7%)	Miniature Poodle – 1 (5.0%)
	Jack Russell – 5 (13.9%)	Pekingese – 2 (10.0%)
	French Bulldog – 4 (11.1%)	Jack Russell – 1 (5.0%)
	Terrier – 3 (8.3%)	Pomeranian – 5 (25.0%)
	Shih Tzu – 2 (5.6%)	Terrier – 3 (15.0%)
	Bichon Frise – 1 (2.8%)	Beagle – 3 (15.0%)
Weight (kg)	< 3 kg – 21 (58.3%)	< 3 kg – 5 (25.0%)
	3–5 kg – 7 (19.4%)	3–5 kg – 5 (25.0%)
	5–10 kg – 5 (13.9%)	5–10 kg – 6 (30.0%)
	10–15 kg – 3 (8.3%)	10–15 kg – 4 (20.0%)

**Table 2:** Echocardiographic measurements and distribution relative to reference ranges in anxious dogs (*n* = 36) and control dogs (*n* = 20)

Cardiac Parameter	Anxious Dogs (*n* = 36)	Control Dogs (*n* = 20)
IVSd (mm)	8.80 ± 0.25 (8.4–9.5)	9.00 ± 0.30 (8.5–9.6)
	Below: 0.0%/Normal: 100.0%/Above: 0.0%	Below: 0.0%/Normal: 100.0%/Above: 0.0%
IVSs (mm)	13.93 ± 2.06 (11.5–17.2)	13.20 ± 1.50 (11.8–15.5)
	Below: 8.3%/Normal: 80.6%/Above: 11.1%	Below: 10.0%/Normal: 90.0%/Above: 0.0%
LVIDd (mm)	38.95 ± 7.99 (22.0–50.3)	36.50 ± 4.00 (30.0–44.0)
	Below: 5.6%/Normal: 61.1%/Above: 33.3%	Below: 0.0%/Normal: 85.0%/Above: 15.0%
LVIDs (mm)	24.49 ± 4.90 (18.5–33.2)	22.80 ± 3.20 (19.0–28.0)
	Below: 0.0%/Normal: 77.8%/Above: 22.2%	Below: 0.0%/Normal: 100.0%/Above: 0.0%
FS (%)	42.14 ± 10.96 (26.4–55.2)	34.50 ± 5.00 (28.0–45.0)
	Below: 0.0%/Normal: 55.6%/Above: 44.4%	Below: 0.0%/Normal: 100.0%/Above: 0.0%
EF (%)	73.61 ± 13.95 (52.0–95.0)	67.50 ± 7.00 (60.0–80.0)
	Below: 16.7%/Normal: 44.4%/Above: 38.9%	Below: 0.0%/Normal: 90.0%/Above: 10.0%
LA/Ao (ratio)	1.47 ± 0.24 (1.0–2.0)	1.20 ± 0.10 (1.0–1.3)
	Below: 0.0%/Normal: 19.4%/Above: 80.6%	Below: 0.0%/Normal: 100.0%/Above: 0.0%

Normal reference ranges applied uniformly to both groups: IVSd 8–11 mm; IVSs 12–16 mm; LVIDd 30–40 mm; LVIDs 18–28 mm; FS 25–45%; EF 55–75%; LA/Ao ≤ 1.3. All control dogs had FS% values within the normal reference range, whereas 44.4% of anxious dogs exceeded this range

*Abbreviations* *FS* fractional shortening, *EF* ejection fraction, *LVIDd/s* left ventricular internal diameter in diastole/systole, *IVSd/s* interventricular septum in diastole/systole *LA/Ao* left atrial-to-aortic root ratio

### Cardiovascular parameter results

As summarized in Table 2, echocardiographic assessments revealed several findings related to cardiac structure and function. In the anxious group, the mean left ventricular internal dimension at end-systole (LVIDs) was 24.49 ± 4.90 mm, the average ejection fraction (EF%) was 73.61 ± 13.95%, and fractional shortening (FS%) averaged 42.14 ± 10.96%. The left atrial-to-aortic root ratio (LA/Ao) had a mean of 1.47 ± 0.24 (range 1.0–2.0).

When compared with uniform reference ranges, several parameters fell outside the suggested limits. LVIDd was above range in 33.3% and below in 5.6% of anxious dogs. FS% exceeded the standard 25–45% range in 44.4%, while LA/Ao was greater than the conventional 1.3 cutoff in 80.6%. These proportions should be interpreted cautiously, as fixed reference values may not fully account for body size differences. In contrast, interventricular septal thickness in diastole (IVSd) was within the normal range in all anxious dogs. A detailed breakdown of deviations is provided in Table 2.The control group showed values largely within normal limits. Mean LVIDd was 36.50 ± 4.00 mm, FS% was 34.50 ± 5.00%, and EF% was 67.50 ± 7.00%, with only minor deviations observed (e.g., 15% of dogs with LVIDd above reference and 10% with EF% above reference). No significant structural or functional abnormalities were detected in this group.

**Fig. 1:**
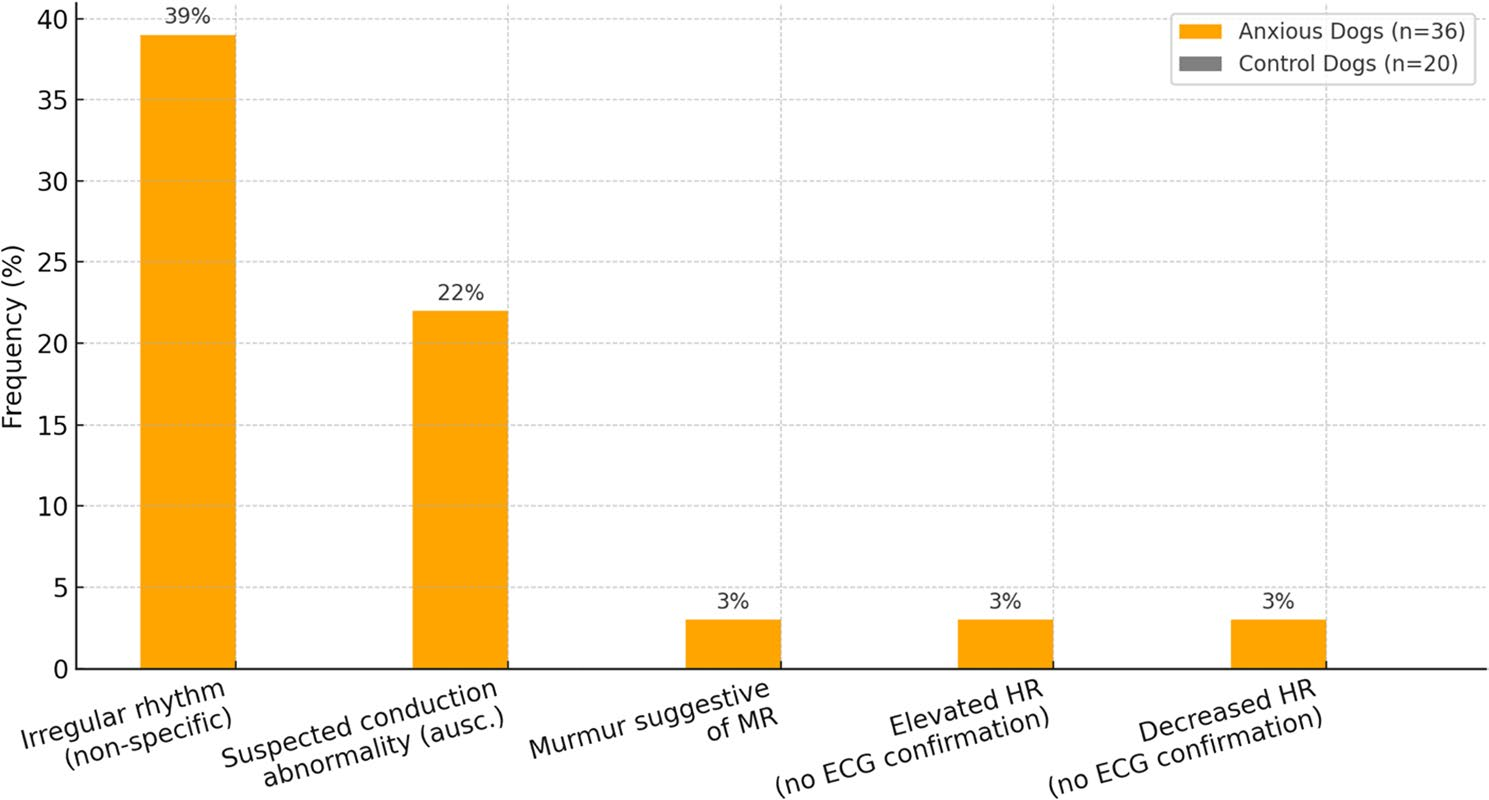
Frequency of detected rhythm and auscultatory irregularities in anxious dogs (*n* = 36) compared with control dogs (*n* = 20). Values reflect findings based on auscultation and echocardiographic observation. Arrhythmia types remain non-specifically categorized due to absence of ECG; no abnormalities were observed in control dogs. Categories are not mutually exclusive; a dog could contribute to more than one finding, and percentages therefore do not sum to 100%

Suspected rhythm irregularities were identified in 47% (17/36) of anxious dogs based on auscultation and echocardiographic observation. The most frequent pattern was a non-specific irregular rhythm (38.9%), followed by findings suggestive of conduction disturbances (22.2%). Less common abnormalities included single cases of tachycardia, bradycardia, and murmurs possibly consistent with mitral regurgitation. As ECG confirmation was not available, these should be regarded as provisional rather than definitive diagnoses. Categories were not mutually exclusive, since some dogs exhibited more than one finding. The distribution of abnormalities is illustrated in Fig. 1.

### Correlation between anxiety and cardiovascular parameters

**Fig. 2:**
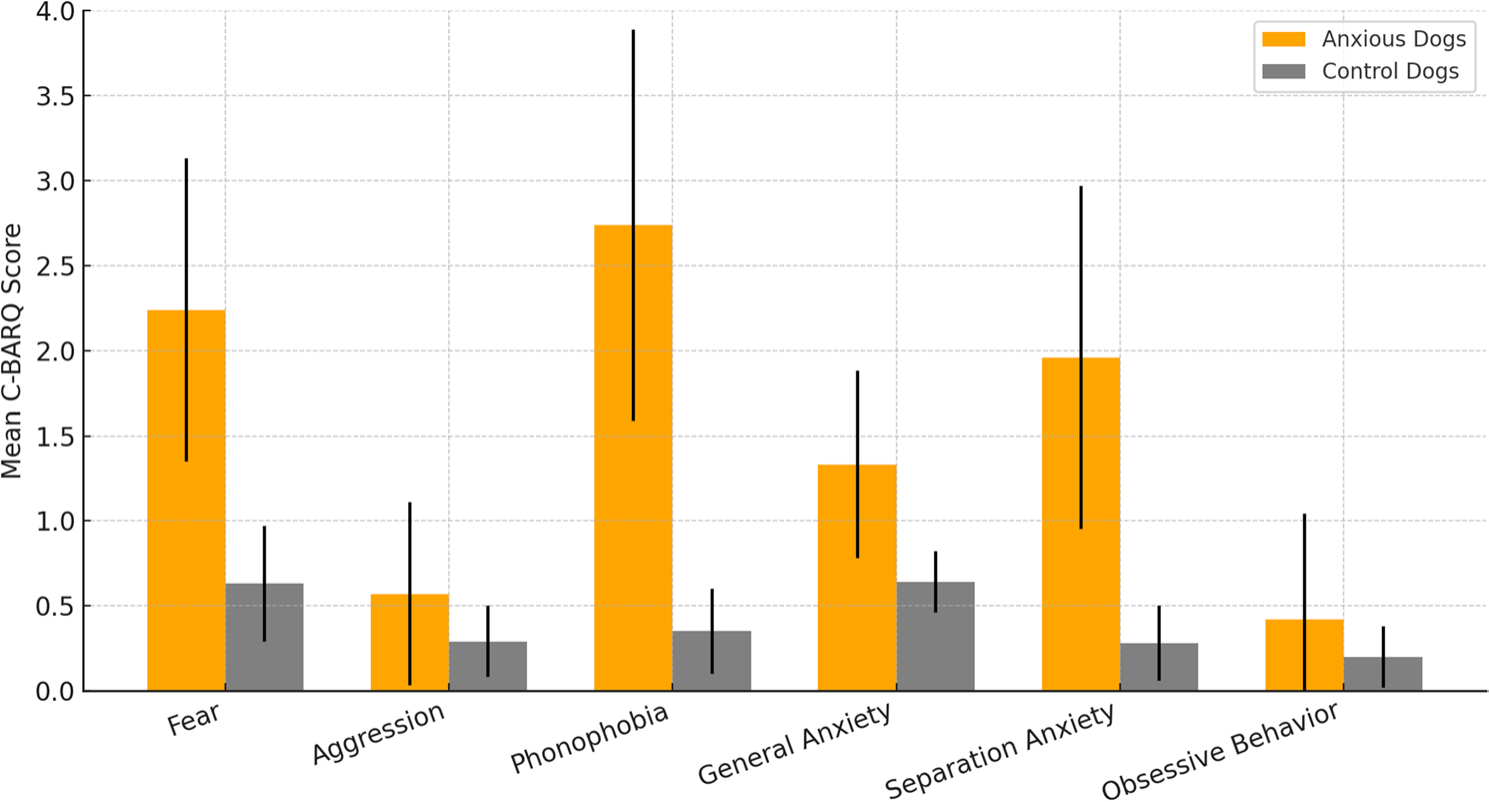
Mean ± standard deviation (SD) of anxiety-related behavioral parameters in anxious dogs (*n* = 36) compared with control dogs (*n* = 20), based on C-BARQ questionnaire results. Control dogs consistently exhibited minimal scores across all domains, whereas anxious dogs showed markedly elevated scores, particularly in phonophobia, fear, and separation anxiety

Statistical analysis revealed significant correlations between specific anxiety subtypes and key echocardiographic parameters in anxious dogs. General anxiety was positively associated with fractional shortening (FS%) (*r* = 0.663, *p* = 0.001, q = 0.004). Phonophobia showed a strong positive correlation with left ventricular internal dimension at end-diastole (LVIDd) (*r* = 0.681, *p* < 0.001, q = 0.004). Although 61.1% of anxious dogs had LVIDd values within the reference range, those with higher phonophobia scores tended toward larger or above-reference diastolic dimensions, which may partly reflect body size variation or measurement variability. Aggression was correlated with the left atrial-to-aortic root ratio (LA/Ao) (*r* = 0.475, *p* = 0.008, q = 0.016); however, because LA/Ao values exceeded 1.3 in most anxious dogs, this association should be regarded as exploratory (Table 3, Fig. 2).By contrast, none of these associations were significant in the control group (all *p* > 0.6, *r* < 0.2), indicating that the observed relationships between anxiety traits and cardiac parameters were specific to anxious dogs.

**Table 3:** Correlation Analyses Related to Anxiety and Cardiac Function. Pearson correlations between anxiety subtypes and echocardiographic parameters in anxious (*n* = 36) and control (*n* = 20) dogs

Anxiety Parameter	Cardiac Parameter	Anxious Dogs (*n* = 36) r [95% CI]	*p*	q	Control Dogs (*n* = 20) r [95% CI]	*p*	q
General Anxiety	FS%	+ 0.663 [0.37, 0.83]	0.001	0.004*	+ 0.112 [− 0.36, 0.54]	0.621	0.78
Phonophobia	LVIDd	+ 0.681 [0.40, 0.84]	< 0.001	0.004*	+ 0.085 [− 0.39, 0.52]	0.734	0.81
Aggression	LA/Ao	+ 0.475 [0.12, 0.72]	0.008	0.016*	+ 0.094 [− 0.38, 0.53]	0.698	0.79

*CI* 95% confidence interval for *r* (calculated via Fisher’s z transform)

q-values reflect FDR correction across all correlations within each table

Heart rate also showed significant associations with multiple cardiac indices in the anxious cases. It correlated strongly with left ventricular free wall thickness in systole (LVFWs) (*r* = 0.833, *p* < 0.001, q = 0.002), FS% (*r* = 0.663, *p* = 0.001, q = 0.006), and EF% (*r* = 0.644, *p* = 0.002, q = 0.008) (Table 4, Fig. 2). A positive correlation was also observed with LVIDd (*r* = 0.681, *p* < 0.001, q = 0.002), although this finding contrasts with physiological expectations, as tachycardia typically reduces diastolic filling. This pattern may therefore reflect confounding by body size, anxiety severity, or measurement variability. Taken together, these results suggest synchronized increases in contractility and myocardial wall thickness in dogs with higher heart rates. In the control group, heart rate correlations with echocardiographic indices were weak and non-significant (all *p* > 0.2).

**Table 4:** Correlation Analyses Related to Anxiety and Cardiac Function. Correlation coefficients between heart rate and echocardiographic parameters, and among cardiac metrics, in anxious (*n* = 36) and control (*n* = 20) dogs

Cardiac Parameter(s)	Anxious (*n* = 36) r [95% CI]	*p*	q	Control (*n* = 20) r [95% CI]	*p*	q
IVSd – Heart Rate	+ 0.709 [0.45, 0.85]	< 0.001	0.002*	+ 0.152 [− 0.34, 0.56]	0.521	0.73
LVIDd – Heart Rate	+ 0.681 [0.40, 0.84]	< 0.001	0.002*	+ 0.189 [− 0.31, 0.58]	0.418	0.70
LVFWs – Heart Rate	+ 0.833 [0.66, 0.92]	< 0.001	0.002*	+ 0.276 [− 0.22, 0.66]	0.231	0.64
LVFWd – Heart Rate	+ 0.772 [0.56, 0.89]	< 0.001	0.002*	+ 0.214 [− 0.28, 0.62]	0.352	0.68
FS% – Heart Rate	+ 0.663 [0.36, 0.83]	0.001	0.006*	+ 0.101 [− 0.37, 0.52]	0.676	0.80
EF% – Heart Rate	+ 0.644 [0.33, 0.82]	0.002	0.008*	+ 0.128 [− 0.35, 0.55]	0.602	0.77
LVIDs – Heart Rate	+ 0.475 [0.12, 0.72]	0.008	0.016*	+ 0.173 [− 0.32, 0.57]	0.462	0.72
LVFWs – LVIDs	+ 0.795 [0.57, 0.90]	< 0.001	0.002*	+ 0.212 [− 0.28, 0.62]	0.359	0.69
LVFWd – LVIDd	+ 0.776 [0.55, 0.89]	< 0.001	0.002*	+ 0.248 [− 0.25, 0.63]	0.278	0.66
LVFWs – LVFWd	+ 0.843 [0.68, 0.92]	< 0.001	0.002*	+ 0.193 [− 0.30, 0.59]	0.407	0.71
FS% – LVFWs	+ 0.759 [0.52, 0.88]	< 0.001	0.002*	+ 0.226 [− 0.27, 0.61]	0.326	0.67
EF% – LVFWs	+ 0.757 [0.52, 0.88]	< 0.001	0.002*	+ 0.201 [− 0.29, 0.60]	0.386	0.70
FS% – EF%	+ 0.987 [0.97, 0.99]	< 0.001	0.002*	+ 0.912 [0.79, 0.97]	< 0.001	0.004*

*CI* 95% confidence interval for *r*. q-values reflect Benjamini–Hochberg FDR across all correlations in this table (per group columns shown for clarity). Asterisks indicate q < 0.05

Further analysis of inter-parameter relationships (Table 5) demonstrated several significant associations in anxious dogs. FS% correlated positively with interventricular septal thickness in systole (IVSs; *r* = 0.401, *p* = 0.048, q = 0.049) and negatively with LVIDs (*r* = − 0.476, *p* = 0.029, q = 0.041). EF% was also negatively correlated with LVIDs (*r* = − 0.452, *p* = 0.037, q = 0.046). LA/Ao correlated significantly with IVSs (*r* = 0.389, *p* = 0.041, q = 0.049), LVIDs (*r* = 0.427, *p* = 0.042, q = 0.049), and FS% (*r* = 0.498, *p* = 0.025, q = 0.036). These findings indicate consistent internal relationships between functional and geometric cardiac parameters in anxious dogs, though interpretation is limited by the use of uniform reference ranges. In the control group, such interrelationships were again weak and non-significant (*r* < 0.2, *p* > 0.5).

**Table 5:** Correlation Analyses Related to Anxiety and Cardiac Function. Interrelationships between cardiac structural and functional indices in anxious (*n* = 36) and control (*n* = 20) dogs

Cardiac Parameter	Correlated Parameter	Anxious (*n* = 36) r [95% CI]	*p*	q	Control (*n* = 20) r [95% CI]	*p*	q
IVSd	IVSs	+ 0.512 [0.17, 0.74]	0.021	0.032*	+ 0.118 [− 0.36, 0.54]	0.612	0.78
LVIDd	IVSd	+ 0.468 [0.11, 0.71]	0.032	0.044*	+ 0.154 [− 0.32, 0.56]	0.498	0.75
FS%	IVSs	+ 0.401 [0.02, 0.67]	0.048	0.049*	+ 0.097 [− 0.37, 0.52]	0.676	0.81
LA/Ao	IVSs	+ 0.389 [0.00, 0.66]	0.041	0.049*	+ 0.082 [− 0.38, 0.51]	0.732	0.82
LVIDd	LVIDs	+ 0.544 [0.21, 0.75]	0.018	0.029*	+ 0.163 [− 0.31, 0.57]	0.472	0.74
FS%	LVIDs	−0.476 [− 0.72, − 0.11]	0.029	0.041*	+ 0.135 [− 0.33, 0.55]	0.556	0.79
EF%	LVIDs	−0.452 [− 0.70, − 0.08]	0.037	0.046*	+ 0.109 [− 0.35, 0.54]	0.645	0.80
LA/Ao	LVIDs	+ 0.427 [0.05, 0.69]	0.042	0.049*	+ 0.121 [− 0.34, 0.55]	0.604	0.78
FS%	LVIDd	+ 0.435 [0.06, 0.70]	0.040	0.049*	+ 0.142 [− 0.33, 0.56]	0.532	0.77
LA/Ao	LVIDd	+ 0.402 [0.02, 0.67]	0.047	0.049*	+ 0.116 [− 0.34, 0.55]	0.618	0.79
LA/Ao	FS%	+ 0.498 [0.15, 0.73]	0.025	0.036*	+ 0.157 [− 0.32, 0.57]	0.487	0.76

Values represent Pearson correlation coefficients (r) with 95% confidence intervals (calculated via Fisher’s z transform), unadjusted p-values, and Benjamini–Hochberg false discovery rate (FDR)–adjusted q-values. Asterisks (*) denote correlations that remained statistically significant after FDR correction (q < 0.05)

*Abbreviations* *HR* heart rate, *FS* fractional shortening, *EF* ejection fraction, *LVIDd/s* left ventricular internal diameter in diastole/systole, *LVFWd/s* left ventricular free wall thickness in diastole/systole, *IVSd/s* interventricular septum in diastole/systole, *LA/Ao* left atrial-to-aortic root ratio

Age also influenced several structural measures. Younger anxious dogs exhibited lower IVSs, with a positive but non-significant correlation between age and IVSs (*r* = 0.291, *p* = 0.084). By contrast, LVFWd and LVFWs were strongly correlated (*r* = 0.843, *p* < 0.001, q = 0.002), reflecting consistent myocardial wall thickening across age groups. These patterns were observed in both anxious and control dogs, although statistical significance was reached only in the anxious cases.

### Anxiety profile distribution and summary

**Fig. 3:**
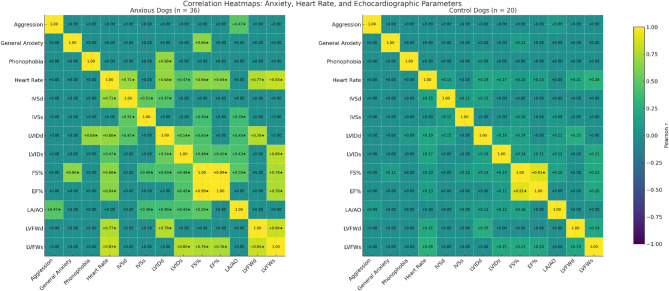
Correlation heatmaps comparing anxious dogs (*n* = 36) and control dogs (*n* = 20). Pearson correlation coefficients (r) are shown for behavioral anxiety subtypes, heart rate, and echocardiographic parameters. Darker shades denote stronger correlations. Asterisks (*) indicate correlations that remained statistically significant after Benjamini–Hochberg false discovery rate (FDR) correction across the family of correlation tests (q < 0.05). Data correspond to Tables 3, 4 and 5

Behavioral anxiety scores in the anxious group (*n* = 36) demonstrated wide variability across domains. Fear-related behaviors were among the most prominent: 33.3% of dogs scored between 2 and 3, while 44.4% scored between 3 and 4, indicating moderate to high levels of fearfulness. Aggression scores, by contrast, were consistently low, with 41.7% of dogs scoring below 1 and 55.6% between 1 and 2; notably, none exceeded a score of 3.Phonophobia showed the greatest variability. About 27.8% of anxious dogs scored between 2 and 3, while 36.1% scored above 4, reflecting heightened sensitivity to loud or sudden noises. General anxiety occurred mainly at low to moderate levels, with 50.0% of dogs scoring between 1 and 2 and 47.2% between 2 and 3. Separation anxiety displayed a more balanced distribution: 41.7% scored between 2 and 3, 22.2% between 1 and 2, and 8.3% between 3 and 4. Obsessive-compulsive behaviors (OCD) were least common, with 63.9% of anxious dogs scoring below 1 and only 5.6% scoring 2 or higher.In contrast, the control group (*n* = 20) consistently showed minimal anxiety-related behaviors. Their mean C-BARQ scores ranged from 0.20 ± 0.18 for obsessive-compulsive behaviors to 0.64 ± 0.18 for general anxiety, confirming their role as a non-anxious comparator group (Table 6; Fig. 3).As illustrated in Fig. 3, phonophobia had the highest mean score among anxious dogs (2.74 ± 1.15), followed by fear (2.24 ± 0.89) and separation anxiety (1.96 ± 1.01). The lowest mean scores were recorded for aggression (0.57 ± 0.54) and obsessive-compulsive behavior (0.42 ± 0.62), consistent with their limited prevalence in this population. Control dogs remained near baseline across all domains. Full categorical distributions are provided in Table 6.

**Table 6:** Distribution of anxiety-related behavioral scores (C-BARQ) across six domains in anxious dogs (*n* = 36), with mean scores for the control group (*n* = 20)

Behavioral Parameter	Category (Anxious Dogs, *n* = 36)	Count	Frequency (%)	Control Group (*n* = 20, Mean ± SD)
Fear	< 1	1	2.8	0.63 ± 0.34
	1–2	6	16.7	
	2–3	12	33.3	
	3–4	17	47.2	
Aggression	< 1	15	41.7	0.29 ± 0.21
	1–2	20	55.6	
	2–3	1	2.8	
	3–4	0	0.0	
Phonophobia	< 1	1	2.8	0.35 ± 0.25
	1–2	4	11.1	
	2–3	10	27.8	
	3–4	21	58.3	
General Anxiety	< 1	1	2.8	0.64 ± 0.18
	1–2	18	50.0	
	2–3	17	47.2	
	3–4	0	0.0	
Separation Anxiety	< 1	2	5.6	0.28 ± 0.22
	1–2	8	22.2	
	2–3	15	41.7	
	3–4	11	30.6	
OCD	< 1	23	63.9	0.20 ± 0.18
	1–2	11	30.6	
	≥ 2 *(upper bin)*	2	5.6	

Bins reflect mean Likert scores (0–4) across items within each domain. For OCD, sparse data were collapsed into ≥ 2

## Discussion

This study explored the association between behavioral anxiety and echocardiographic parameters in dogs, revealing measurable cardiac changes linked to chronic emotional stress. General anxiety was significantly correlated with increased fractional shortening (FS%), suggesting a hyperdynamic systolic state likely driven by sympathetic activation, while phonophobia was associated with relatively greater left ventricular diastolic dimensions (LVIDd) and aggression with elevated left atrial-to-aortic root ratios (LA/Ao). These findings parallel human data linking emotional stress to atrial remodeling and arrhythmia risk [11, 12]. In addition, both FS% and ejection fraction (EF%) showed negative correlations with systolic chamber size (LVIDs), consistent with expected physiological relationships, and heart rate correlated strongly with myocardial wall thickness, FS%, and EF%, indicating coordinated contractile changes under heightened arousal (Table 5, Fig. 2). By contrast, none of these associations were observed in the control group, where correlations remained weak and non-significant, reinforcing that the cardiac–behavioral relationships were specific to anxious dogs.

The association between general anxiety and elevated fractional shortening (FS%) observed in this study suggests a hyperdynamic myocardial state consistent with chronic sympathetic nervous system activation. Similar findings are well documented in human psychocardiology, where persistent emotional stress promotes catecholamine release, heightened myocardial contractility, and progressive structural adaptations. The increased FS% in anxious dogs therefore likely reflects autonomic imbalance–mediated cardiac hyperkinesis. The positive correlation between phonophobia and left ventricular diastolic dimension (LVIDd) may provide additional insight, as noise-sensitive individuals exhibited relatively larger diastolic chambers despite the group mean remaining below reference values—likely due to small body size. This pattern could represent early-stage remodeling associated with increased preload or stress-induced dilation. Likewise, the association between aggression and higher left atrial-to-aortic root ratios (LA/Ao) may indicate early atrial remodeling in response to repeated behavioral arousal, though causal inference cannot be drawn from this cross-sectional design. Finally, the strong correlations between heart rate and multiple echocardiographic indices (LVFWs, FS%, EF%) reinforce the biological plausibility of anxiety-driven cardiac remodeling, particularly since these relationships were absent in the control group.

Our findings partially align with existing veterinary literature but differ in emphasizing chronic, naturally occurring emotional stress rather than acute or pharmacological stressors. Most previous work in veterinary cardiology has examined transient stress responses or drug effects, whereas this study evaluated prolonged anxiety in client-owned dogs under real-world conditions. Pradelli et al. (2014) showed that acute emotional stress during echocardiographic handling transiently elevated aortic peak velocity in Boxer dogs, reflecting a hyperdynamic response, which parallels our observation of elevated fractional shortening (FS%; mean 42.14 ± 10.96%), exceeding reference limits in 44.4% of anxious dogs and suggesting sustained sympathetic activation [13]. In contrast, Fries et al. (2019) reported no significant changes in FS% or ejection fraction (EF%) after trazodone administration in healthy cats, highlighting the importance of excluding medicated dogs in our study and underscoring that the higher FS% in anxious dogs is more plausibly explained by chronic anxiety than pharmacological effects [14]. Neuroimaging work by Xu et al. (2023) further supports this interpretation by demonstrating disrupted connectivity in brain regions regulating emotion (e.g., amygdala, hippocampus) in anxious dogs, reinforcing the link between emotional dysregulation and physiological outcomes [15]. Similarly, Wang et al. (2025) identified early myocardial impairment under radiation-induced stress in Beagles using speckle-tracking echocardiography; unlike those dogs, our anxious cases displayed increased FS% and EF%, suggesting that emotional stress may initially induce compensatory cardiac responses before progressing to maladaptive remodeling depending on stressor chronicity [16]. Taken together, these comparisons highlight the novelty and clinical significance of our work, providing evidence that naturally occurring chronic emotional stress can drive subclinical cardiovascular remodeling in dogs, consistent with mechanisms established in human psychocardiology [17–20].

This study has important implications for veterinary cardiology and behavioral medicine. Traditionally, canine anxiety is addressed through behavioral modification, environmental enrichment, and pharmacological treatment, but our findings suggest that chronic anxiety may also act as a driver of early-stage cardiac remodeling, even in dogs without overt cardiovascular disease. Increases in fractional shortening (FS%), left ventricular internal dimension in diastole (LVIDd), and left atrial-to-aortic root ratio (LA/Ao) observed in anxious dogs indicate that sustained emotional stress can promote subclinical cardiovascular adaptations [21]. Although these parameters generally remained within reference ranges, they may represent compensatory states that precede pathological remodeling. Elevated FS%, present in 44.4% of anxious dogs, likely reflects sympathetic dominance which, in predisposed individuals or breeds, could progress to hypertrophy or diastolic dysfunction. By contrast, control dogs remained within normal limits, reinforcing that these changes were specific to anxiety rather than incidental variation.

Integrating standardized behavioral screening tools such as the validated C-BARQ into routine veterinary practice may therefore support early identification of dogs at risk, particularly those with high scores for general anxiety, phonophobia, or aggression. Such individuals could benefit from proactive cardiac monitoring, including serial echocardiography, to enable timely intervention. Importantly, clinicians should interpret echocardiographic findings in the context of behavioral status: functional hyperkinesis or mild atrial enlargement in reactive dogs should not be automatically attributed to primary cardiac pathology [3]. Recognizing the influence of emotional stress on cardiac function can improve diagnostic accuracy, guide treatment decisions, and help avoid unnecessary pharmacological interventions.Ultimately, these findings support a more integrated veterinary approach that combines behavioral and cardiac assessments. Such a holistic model could facilitate earlier, targeted interventions and improve both emotional and cardiovascular health in companion animals.

Despite providing novel insights, this study has several limitations. Although a control group of clinically healthy, non-anxious dogs was included, it was not fully matched to the anxious cases in age, body size, or breed distribution. These differences may partly explain some echocardiographic findings and limit attribution solely to anxiety. Breed predispositions—particularly in Shiba Inus and Maltese—may also have influenced baseline cardiac metrics. Reliance on owner-reported C-BARQ data, while validated, introduces subjectivity and potential reporting bias. The relatively small sample size (36 anxious, 20 controls) limits statistical power and generalizability, while the cross-sectional design prevents causal inference. In addition, multicollinearity among anxiety subtypes was not formally assessed and may have contributed to overlapping variance.Interpretation of echocardiographic parameters against fixed reference ranges is another constraint, as more than half of the anxious dogs weighed < 3 kg. Size-related misclassification is therefore possible, particularly for LVIDd, and future work should employ size-indexed indices (e.g., LVIDDN) to improve accuracy. An unexpected positive correlation between heart rate and LVIDd also emerged, which runs counter to physiological expectations. This paradox may reflect allometric scaling in very small dogs, confounding by anxiety severity, or measurement variability in fast heart rates, and should be regarded as exploratory pending replication in larger, size-matched case–control groups.Finally, the absence of electrocardiographic (ECG) data represents a significant limitation. Although rhythm abnormalities were suspected in nearly half of the anxious dogs, their classification remained provisional, based only on auscultation and echocardiographic impressions. Future studies should address these limitations by enrolling larger, multicenter case–control groups with matched controls, using longitudinal designs to explore temporal relationships, incorporating objective stress biomarkers (e.g., heart rate variability, serum cortisol, salivary alpha-amylase), and applying advanced imaging modalities such as tissue Doppler or speckle-tracking echocardiography. Interventional studies assessing whether behavioral therapy or anxiolytic medications can mitigate anxiety-related cardiac changes would also provide clinically relevant guidance for optimizing both emotional and cardiovascular health in dogs.

## Conclusion

This study provides novel evidence that chronic behavioral anxiety in dogs is associated with measurable alterations in cardiac structure and function, including elevated fractional shortening, increased left ventricular dimensions, and higher LA/Ao ratios. These findings suggest that persistent emotional stress may promote early, subclinical cardiac remodeling even in the absence of overt heart disease. Recognizing anxiety as both a behavioral and cardiovascular condition underscores the importance of incorporating standardized behavioral assessments into routine veterinary care.Although limited by sample size and the absence of fully matched controls for breed and body size, the results highlight the need for larger, longitudinal studies incorporating objective stress biomarkers to clarify causality and disease progression. Such work could refine early diagnostic strategies and support preventive interventions. Ultimately, addressing anxiety as a systemic condition may improve both emotional well-being and long-term cardiovascular health in companion dogs.

## Supplementary Information

Supplementary Material 1.

## Data Availability

The datasets used and/or analyzed during the current study are available from the corresponding author (Dr. Mohammad Molazem) on reasonable request.

## References

[1] Salonen M, Sulkama S, Mikkola S, Puurunen J, Hakanen E, Tiira K, et al. Prevalence, comorbidity, and breed differences in canine anxiety in 13,700 Finnish pet dogs. Sci Rep. 2020;10:2962. 32139728 10.1038/s41598-020-59837-z PMC7058607

[2] Sacoor C, Marugg JD, Lima NR, Empadinhas N, Montezinho L. Gut-brain axis impact on canine anxiety disorders: new challenges for behavioral veterinary medicine. Vet Med Int. 2024;2024:2856759. 38292207 10.1155/2024/2856759 PMC10827376

[3] Wormald D, Lawrence AJ, Carter G, Fisher AD. Reduced heart rate variability in pet dogs affected by anxiety-related behaviour problems. Physiol Behav. 2017;168:122–7. 27838312 10.1016/j.physbeh.2016.11.003

[4] Richter H, Kircher PR, Joerger FB, Bruellmann E, Dennler M. Assessment of myocardial perfusion at rest and during stress using dynamic first-pass contrast-enhanced magnetic resonance imaging in healthy dogs. Front Vet Sci. 2018;5:211. 30234137 10.3389/fvets.2018.00211 PMC6131641

[5] Koskela A, Törnqvist H, Somppi S, Tiira K, Kykyri V-L, Hänninen L, et al. Behavioral and emotional co-modulation during dog–owner interaction measured by heart rate variability and activity. Sci Rep. 2024;14:25201. 39448721 10.1038/s41598-024-76831-x PMC11502769

[6] Pourghasemi Z, Norouzi N, Safari N, Khakpour H, Keypoori D, Shams F, et al. Prevalence of congenital heart diseases in dogs in Tehran, Iran: a retrospective study from 2013 to 2023. Vet Med Int. 2025;2025:2994461. 40008365 10.1155/vmi/2994461 PMC11858710

[7] Lucina SB, Sarraff AP, Wolf M, Silva VB, Sousa MG, Froes TR. Congenital heart disease in dogs: a retrospective study of 95 cases. Top Companion Anim Med. 2021;43:100505. 33346164 10.1016/j.tcam.2020.100505

[8] Gugjoo MB, Saxena AC, Hoque M, Zama MMS. M-mode echocardiographic study in dogs. Afr J Agric Res. 2014;9:387–96.

[9] Saini N, Uppal SK, Randhawa CS. Echocardiographic parameters and indices in healthy Labrador retriever dogs. Isr J Vet Med. 2017;72:28–32.

[10] DE MADRON ED, Chetboul V, Bussadori C. Clinical echocardiography of the dog and cat. Elsevier Health Sciences; 2015.

[11] Wang Z, Qin H, Chen G, Dai Y, Cai Y, Cheng X, et al. Anxiety is associated with increased risk for atrial cardiopathy. Acta Neurol Belg. 2020;120:1383–8. 32193730 10.1007/s13760-020-01335-0

[12] Wu H, Li C, Li B, Zheng T, Feng K, Wu Y. Psychological factors and risk of atrial fibrillation: a meta-analysis and systematic review. Int J Cardiol. 2022;362:85–92. 35618103 10.1016/j.ijcard.2022.05.048

[13] Pradelli D, Quintavalla C, Crosta MC, Mazzoni L, Oliveira P, Scotti L, et al. The influence of emotional stress on Doppler-derived aortic peak velocity in boxer dogs. J Vet Intern Med. 2014;28:1724–30. 25312007 10.1111/jvim.12434 PMC4895642

[14] Fries RC, Kadotani S, Vitt JP, Schaeffer DJ. Effects of oral trazodone on echocardiographic and hemodynamic variables in healthy cats. J Feline Med Surg. 2019;21:1080–5. 30499766 10.1177/1098612X18814565 PMC10814276

[15] Xu Y, Christiaen E, De Witte S, Chen Q, Peremans K, Saunders JH, et al. Network analysis reveals abnormal functional brain circuitry in anxious dogs. PLoS ONE. 2023;18:e0282087. 36920933 10.1371/journal.pone.0282087 PMC10016658

[16] Wang Z-Y, Huang L, Li L-Q, Zhang C-Q, Guo L-Y, Liu Y-N, et al. Quantitative evaluation of radiation-induced heart disease in beagle dogs by speckle tracking echocardiography. BMC Cardiovasc Disord. 2025;25:199. 40108535 10.1186/s12872-025-04636-5 PMC11924760

[17] Park G, Thayer JF. From the heart to the mind: cardiac vagal tone modulates top-down and bottom-up visual perception and attention to emotional stimuli. Front Psychol. 2014;5:278. 10.3389/fpsyg.2014.00278 PMC4013470 24817853

[18] Chen X, Xu L, Li Z. Autonomic neural circuit and intervention for comorbidity anxiety and cardiovascular disease. Front Physiol. 2022;13:852891. 35574459 10.3389/fphys.2022.852891 PMC9092179

[19] Rosenblum S, Rab SL, Admon R. Dynamics in physiological acute stress response trajectories: uncovering latent variability. BMC Psychiatry. 2025;25:361. 40211213 10.1186/s12888-025-06807-2 PMC11987242

[20] Grol M, De Raedt R. The link between resting heart rate variability and affective flexibility. Cogn Affect Behav Neurosci. 2020;20:746–56. 32462431 10.3758/s13415-020-00800-w

[21] Avalos-Borges EE, Acevedo-Arcique CM, Segura-Correa JC, Jiménez-Coello M, Garg NJ, Ortega-Pacheco A. Echocardiographic documentation of dilated cardiomyopathy development in dogs naturally infected with trypanosoma cruzi. Animals. 2024;14:1884. 38997996 10.3390/ani14131884 PMC11240442

