# Supplementary material for: The impact of anxiety on canine heart function: a study using echocardiographic techniques
Source: BMC Vet Res. 2025 Oct 14;21:605. doi: 10.1186/s12917-025-05074-3 (PMC12522505; doi:10.1186/s12917-025-05074-3)
Supplement: Supplementary file 1 — Supplementary Material 1. [file 12917_2025_5074_MOESM1_ESM.docx]

**
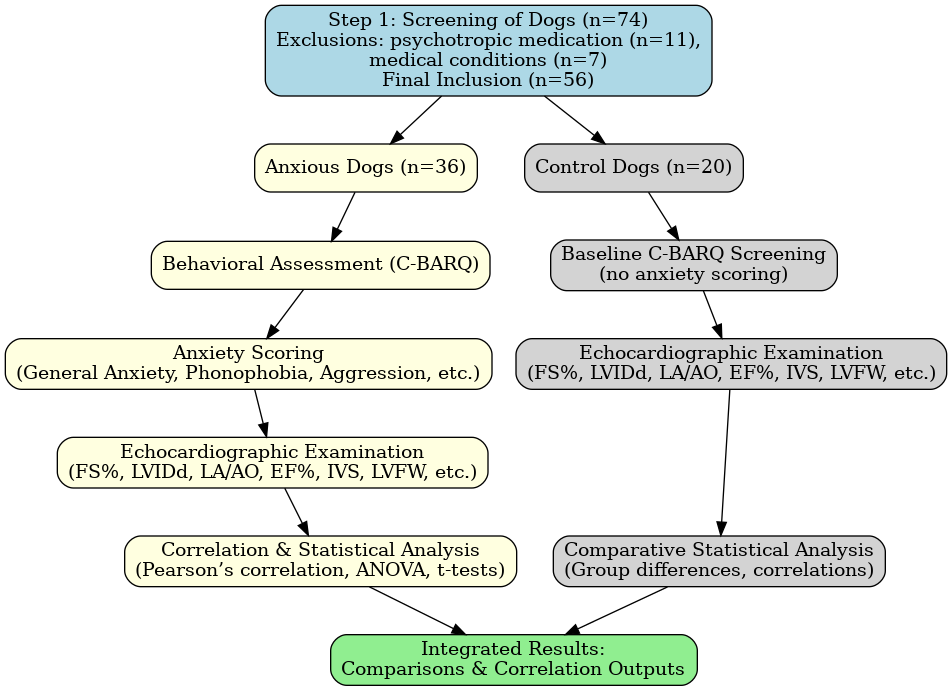
**

**Supplementary Figure S1. Flowchart of study design and sample allocation.**A total of 74 dogs were screened, of which 18 were excluded (psychotropic medication, n = 11; medical conditions, n = 7). This yielded 36 anxious dogs who met the inclusion criteria. In parallel, 20 clinically healthy dogs were recruited as controls. **C-BARQ was administered in both groups; in controls, it was used only to confirm low scores across domains.** Both groups subsequently underwent echocardiographic evaluation and statistical analyses (Tables 1–6; Figures 1–3)
